# Genome-Wide Identification and Characterization of the *CCT* Gene Family in Rapeseed (*Brassica napus* L.)

**DOI:** 10.3390/ijms25105301

**Published:** 2024-05-13

**Authors:** Liyiqi Yu, Jichun Xia, Rujiao Jiang, Jiajia Wang, Xiaolong Yuan, Xinchao Dong, Zhenjie Chen, Zizheng Zhao, Boen Wu, Lanlan Zhan, Ranfeng Zhang, Kang Tang, Jiana Li, Xinfu Xu

**Affiliations:** 1 College of Agronomy and Biotechnology, Southwest University, Beibei, Chongqing 400715, China; yiqibetter99@163.com (L.Y.); jichunxia25@163.com (J.X.); galaxy_yuan@foxmail.com (R.J.); wjjsue99@163.com (J.W.); 13935581923@163.com (X.Y.); xincdone@gmail.com (X.D.); 18198383979@163.com (Z.C.); zhaozz1003@163.com (Z.Z.); 13568525303@139.com (B.W.); zl14375920@163.com (L.Z.); m15501903283@163.com (R.Z.); t52642024@163.com (K.T.); ljn1950@swu.edu.cn (J.L.); 2 Academy of Agricultural Sciences, Southwest University, Beibei, Chongqing 400715, China

**Keywords:** abiotic stress, *Brassica napus*, *CCT* gene family, expression analysis, *PRR* subfamily

## Abstract

The *CCT* gene family is present in plants and is involved in biological processes such as flowering, circadian rhythm regulation, plant growth and development, and stress resistance. We identified 87, 62, 46, and 40 *CCTs* at the whole-genome level in *B. napus*, *B. rapa*, *B. oleracea*, and *A. thaliana*, respectively. The *CCTs* can be classified into five groups based on evolutionary relationships, and each of these groups can be further subdivided into three subfamilies (*COL*, *CMF,* and *PRR*) based on function. Our analysis of chromosome localization, gene structure, collinearity, cis-acting elements, and expression patterns in *B. napus* revealed that the distribution of the 87 *BnaCCTs* on the chromosomes of *B. napus* was uneven. Analysis of gene structure and conserved motifs revealed that, with the exception of a few genes that may have lost structural domains, the majority of genes within the same group exhibited similar structures and conserved domains. The gene collinearity analysis identified 72 orthologous genes, indicating gene duplication and expansion during the evolution of *BnaCCTs*. Analysis of cis-acting elements identified several elements related to abiotic and biotic stress, plant hormone response, and plant growth and development in the promoter regions of *BnaCCTs*. Expression pattern and protein interaction network analysis showed that *BnaCCTs* are differentially expressed in various tissues and under stress conditions. The *PRR* subfamily genes have the highest number of interacting proteins, indicating their significant role in the growth, development, and response to abiotic stress of *B. napus*.

## 1. Introduction

The *CCT* gene family, extensively researched across various plant species, plays a crucial role in multiple biological functions. Each gene within this family features a CCT domain, which is a conserved sequence of 43 amino acids located at the carboxyl terminus of three specific proteins in *A. thaliana*: CONSTANS (CO), CO-LIKE (COL), and TIMING OF CAB1 (TOC1). These proteins are predominantly associated with flowering plants [1,2]. Based on the variations within their conserved domains, *CCT* family genes are organized into three distinct subfamilies, namely CONSTANS-Like (*COL*), CCT Motif Family (*CMF*), and Pseudo Response Regulator (*PRR*). The *COL* subfamily possesses both the B-BOX and CCT domains, whereas the *PRR* subfamily includes the REC and CCT domains. In contrast, the *CMF* subfamily comprises solely the CCT domain [2,3,4]. 

Cockram et al., through their phylogenetic analysis of the CMF, COL, and PRR proteins in the Poaceae family, discovered that the *COL* genes experienced a loss of the B-BOX domain. This loss led to the emergence of the *CMF* subfamily, highlighting a significant evolutionary adaptation [4]. These domain variations further enhance the functional diversification of the *CCT* family.

Research has shown that the genes of the *CCT* family regulate various plant functions, including flowering time, circadian rhythms, and photoperiod responses [5]. Additionally, they influence responses to abiotic stressors, including drought, salinity, cold, and heat, through hormone signaling pathways [6,7]. Therefore, the *CCT* family is pivotal in regulating plant growth, development, and resilience to stress. The *A. thaliana* base genome has 40 members in the *CCT* family, with the *CO* gene being the first identified. It promotes flowering under long-day conditions, and mutants of this gene exhibit late-flowering traits [8]. The *TOC1* gene regulates flowering photoperiods through circadian rhythms, while the *COL1* gene significantly contributes to resistance against abiotic stressors such as salt, drought, and extreme temperatures [9]. Rice has 41 *CCTs*, with recent studies identifying at least 18 members involved in the flowering process. Three newly discovered members, *OsCCT22*, *OsCCT38*, and *OsCCT41*, regulate the heading stage. These genes exhibit a dual regulatory role dependent on photoperiod conditions. Under long-day conditions, *OsCCT22*, *OsCCT38*, and *OsCCT41* act to inhibit flowering, thereby delaying reproductive development until more favorable environmental conditions prevail. Conversely, under short-day conditions, these same genes facilitate the promotion of flowering. Additionally, another gene, *OsCCT03*, has been identified as a unique regulator within this family, possessing the ability to promote flowering irrespective of day length [10]. In *Medicago truncatula*, *CCT* family genes also contribute to plant resistance against non-biological stresses by regulating the abscisic acid (ABA) pathway [6]. 

*B. napus*, commonly known as rapeseed, is recognized globally as a vital oilseed crop. It holds a pivotal position in the agricultural sector due to its significant contribution to the global supply of vegetable oil [11]. Flowering is a pivotal phase in the life cycle of plants, signifying the shift from vegetative growth to reproductive development. Precisely timed flowering is essential for optimizing both the growth period and the maturation process of rapeseed. In various ecological regions, the selection of rapeseed varieties with appropriate maturation periods is crucial, as the length of the flowering period directly determines the potential cultivation areas and the extent of acreage that can be cultivated. Ensuring that flowering occurs at the optimal time is vital for enhancing plant yields by allowing the plants to circumvent environmental conditions that are not conducive to growth [12,13,14,15,16]. Additionally, during its developmental cycle, rapeseed is exposed to multiple abiotic stresses, such as drought, frost, and salinity, which can severely impact the process of oil synthesis and ultimately reduce oil yield [17]. 

The *CCT* gene family has been shown to play diverse and important roles in plant development, circadian rhythms, and tolerance to abiotic stresses in multiple crops [8,18,19,20]. Ye identified that the expression of *CCT*s was downregulated under drought conditions, enhancing drought resistance through mutant deletion. Furthermore, overexpression of *OsTIFY1a*, a *COL* subfamily gene, may improve rice’s drought resilience [21]. Zheng employed quantitative real-time PCR, revealing significantly higher *CO* gene expression in the leaves and buds of early-flowering rapeseed varieties compared to late-flowering ones [22]. Guo discovered that the *BnaA10.CO* allele gene, through consecutive backcrossing, could advance the flowering phase of semi-winter rapeseed [23]. We found that most studies on the *CCT* gene family in rapeseed have focused on the regulation of flowering by the *CO* gene. There is limited research on other subfamilies, and relatively little research on the role of *CCTs* in salt tolerance, drought, high and low temperature stresses, etc., in rapeseed. Therefore, studying the *CCT* gene family in *B. napus* can aid in understanding the molecular mechanisms of environmental stress responses. This is crucial for improving rapeseed yield under varying environmental conditions.

## 2. Results

### 2.1. Identification, Analysis, and Chromosomal Localization of the BnaCCTs

We applied the Hidden Markov Model (HMM), utilizing the specific profile for the CCT domain, identified as PF06203 in the Pfam database [24], to systematically explore the *B. napus* genome. This rigorous analysis led to the identification of 87 distinct *BnaCCTs* (Table S1). The proteins encoded by these genes, termed *BnaCCTs*, exhibit a diverse range of structural features. Their lengths vary substantially, from as few as 96 amino acids in *BnaCCT50* up to 861 amino acids in *BnaCCT25*. Similarly, the molecular weights of these proteins range from a minimal 11.3 kDa in *BnaCCT50* to a significant 94.7 kDa in *BnaCCT25*. Additionally, the predicted isoelectric points of these proteins also show considerable variation, ranging from 4.4 in proteins such as *BnaCCT16* and *BnaCCT38*, up to 10.53 in *BnaCCT03*. According to the Wolf database [25], subcellular localization predictions indicate that 65 members are localized to the nucleus, 17 to the chloroplasts, *BnaCCT12* and *BnaCCT83* to the cytoplasm, *BnaCCT25* and *BnaCCT82* to the plasma membrane, and *BnaCCT47* is localized within the mitochondria.

**Figure 1:**
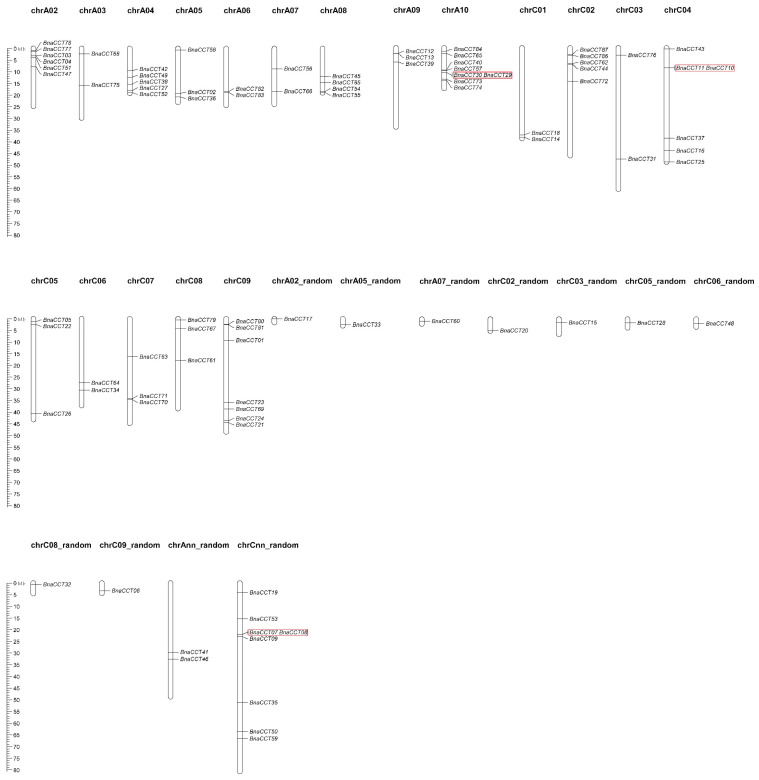
Chromosomal mapping of potential *BnaCCTs* is depicted. This mapping details the specific locations of these genes on individual chromosomes, with each chromosome number prominently displayed above its corresponding chromosome. Furthermore, the lengths of these chromosomes are meticulously indicated in megabases (Mb). The red box highlights genes that represent tandemly repeated genes.

There are 87 *BnaCCTs* in total, with 68 located on 18 of the 19 chromosomes of *B. napus*. The remaining 19 genes were not precisely located in the B. napus genome. Figure 1 shows variation in the number of *BnaCCTs* among various chromosomes, ranging from 1 to 8. Chromosome A10 and the Cnn sequence contain the most genes, each with eight *BnaCCTs* found on various chromosomes: seven on chromosome C9, six on both chromosomes C4 and A2, five on chromosome C5, four on each of chromosomes A4 and A8, and three on each of the chromosomes A9, A5, C7, and C8. Chromosomes A3, A6, A7, C1, C3, and the Ann each contain two *BnaCCTs*. The majority of sequences (A2r, A5r, A7r, C2r, C3r, C5r, C6r, C8r, C9r) only contain one *BnaCCT* gene. The tandem repeat sequences *BnaCCT29/BnaCCT30*, *BnaCCT11/BnaCCT10*, and *BnaCCT07/BnaCCT08* have been identified.

### 2.2. Evolution and Collinearity Analysis of the CCTs in B. napus and Other Related Species

**Figure 2:**
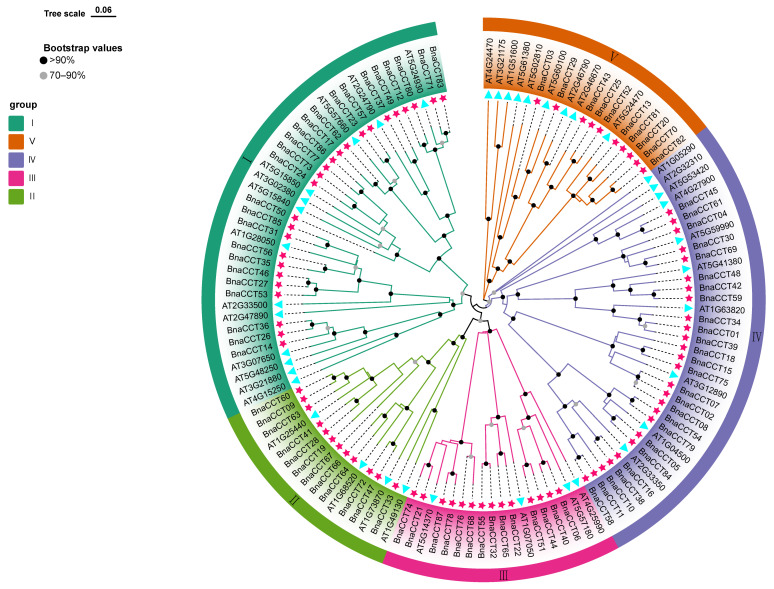
Phylogenetic trees of CCT proteins in *A. thaliana* and *B. napus*. A comprehensive phylogenetic analysis was performed on CCT proteins extracted from A. thaliana and B. napus. Utilizing the maximum likelihood method, this study analyzed 127 protein sequences from both species to ensure a statistically robust framework, enhanced by 1000 bootstrap replicates to verify the consistency of the findings. The figure effectively segregates the proteins into distinct groups, which are visually represented through uniquely color-coded lines for clear differentiation. Specifically, the CCT proteins of *B. napus* are denoted by red stars, while those from *A. thaliana* are indicated by blue triangles.

To elucidate the molecular evolution and establish phylogenetic relationships within the *CCT* gene family, we selected *CCTs* from three *Brassica* species and *A. thaliana* (Table S1). The number of *CCTs* identified were as follows: 87 for *B. napus*, 40 for *A. thaliana*, 63 for *B. rapa*, and 47 for *B. oleracea*. This enumeration forms the foundational dataset for our phylogenetic inquiry. Subsequently, phylogenetic trees were constructed to map the evolutionary relationships of the *CCTs* from *A. thaliana* and *B. napus* (Figure 2). According to the findings derived from the analysis of the constructed phylogenetic tree, *CCTs* were categorized into five groups based on parsimony (I, II, III, IV, V). Group I had the highest number of members, totaling 38 (13 for *A. thaliana*, 25 for *B. napus*) and accounting for 29.9% of the total gene members. Group IV had 36 members, comprising 28.3% of the total members, with 10 for *A. thaliana* and 26 for *B. napus*. Meanwhile, groups II, III, and V had 16, 18, and 19 members, respectively.

**Figure 3:**
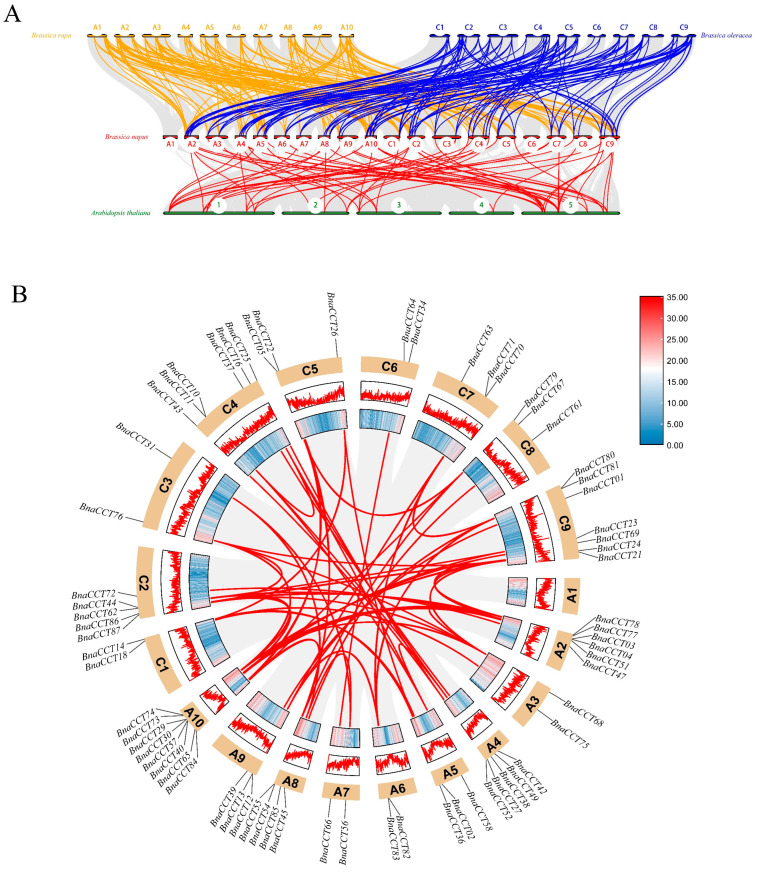
Colinearity analysis of the *BnaCCTs* within and across species of *B. napus*. (**A**) Homology of *CCTs* between *B. napus*, *B. rapa*, *B. oleracea*, and *A. thaliana*. Gray lines delineate the collinear blocks shared among the genomes of these species, emphasizing the structured genomic architecture. Red lines specifically highlight the *CCT* gene pairs that are collinear, underscoring their evolutionary conservation across different species. (**B**) Interchromosomal relationships of *BnaCCT*s. This section visualizes the common genomic blocks as represented by gray lines, which denote regions of shared sequence homology. The red lines are used to specifically point out duplicated *CCT* gene pairs within these blocks, indicating instances of gene replication and divergence that contribute to the genetic complexity and adaptive capacity of *B. napus*.

In order to expand our knowledge of the phylogenetic relationships and developmental behaviors within the *CCT* gene family, a collinearity analysis was conducted on *CCTs* across several species, including *A. thaliana*, *B. rapa*, and *B. oleracea* (Figure 3A). The analysis revealed 74 homologous gene pairs linking *B. napus* with *A. thaliana*. Furthermore, the analysis extended to compare *B. napus* with *B. rapa* and *B. oleracea*, revealing a significant number of homologous pairs: 159 and 146, respectively. Out of these genes, 56 are collinear between *B. napus* and other species (Table S2), including *BnaCCT43*, *BnaCCT70*, *BnaCCT81*, and *BnaCCT82*, which have shown remarkable conservation over time. This conservation highlights their pivotal roles in shaping the genetic trajectory of the *CCT* gene family, suggesting that these genes have not only survived selective pressures but also retained key functional and structural characteristics beneficial to the adaptation and survival of these plants [3,4].

Figure 3B shows that out of the 68 *BnaCCTs* on the 18 chromosomes of *B. napus*, 72 pairs of *CCTs* have undergone large segment duplication. A detailed investigation was carried out to evaluate the evolutionary selection pressure acting on these gene pairs. The rates of nonsynonymous (Ka) and synonymous (Ks) substitutions, as well as the Ka/Ks ratios, were determined for 72 gene pairs (Table S3). The analysis conducted on the Ka/Ks ratios for the gene pairs under study demonstrated that all pairs maintained ratios below the threshold of one. This uniformity across the dataset strongly indicates that these genes have undergone consistent purifying selection. Such a trend in the Ka/Ks ratios typically reflects evolutionary pressures that favor the elimination of deleterious mutations, thereby preserving the functional integrity of the genes. It is worth nothing that the Ka/Ks ratio for the gene pair *BnaCCT58/BnaCCT11* did not conform to model parameters, possibly due to high sequence divergence and considerable evolutionary distance. Using the formula T=Ks÷2r (where r is being considered as 1.5 × 10^−8^) [26], we computed the time of occurrence of large segment duplication for the 72 gene pairs. Table S3 shows that the gene pair *BnaCCT03/BnaCCT29* represents the earliest instance of large segment duplication, occurring approximately 16.69 million years ago (Mya), while the large segment duplication event for *BnaCCT39/BnaCCT01* took place most recently, around 0.35 Mya.

### 2.3. Gene Structure, Conservative Motif Analysis, and Domain Analysis

**Figure 4:**
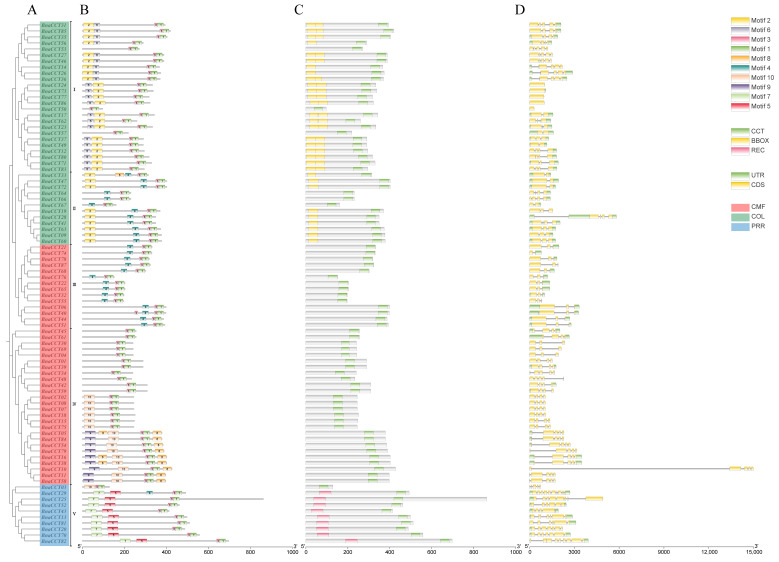
Phylogenetic relationships, gene structure, and conserved motifs of BnaCCT proteins. (I–V): *BnaCCTs* have been organized into five groups based on a previously established phylogenetic tree. (**A**) The phylogenetic tree for these proteins is constructed using domain-specific data derived from *B. napus* CCT proteins. Within this tree, proteins from the *CMF* family are highlighted in red, those from the *COL* family in green, and members of the *PRR* family in blue, effectively categorizing them based on evolutionary lineage. (**B**) The motif composition of the *B. napus* CCT proteins is carefully laid out, with each motif distinctly numbered from 1 to 10 and represented by boxes in varied colors, facilitating easy identification of structural similarities and differences. (**C**) Domain diagrams for the predicted *BnaCCTs* are provided, illustrating the CCT domain in green, the B-BOX domain in yellow, and the REC domain in pink, which delineate the functional components inherent to each protein family. (**D**) Detailed exon–intron structures of the *BnaCCTs* are shown, where untranslated regions are identified by green boxes, exons by yellow boxes, and the intervening sequences, or introns, by black lines, thereby mapping the genetic architecture that supports the diverse functions of these genes.

We developed a phylogenetic tree of *BnaCCTs* (Figure 4A). Drawing from the classifications in a previously established phylogenetic tree (Figure 2), *BnaCCTs* have been organized into five groups (I through V). This categorization aids in furthering our comprehension of the evolution of the *BnaCCTs*. We analyzed and identified the amino acid sequence of proteins from *BnaCTTs* (Figure 4B), domain distribution (Figure 4C), and gene structure (Figure 4D) using the TBtools tool for visualization [27]. According to the included domains (Figure 4C), 87 *BnaCCTs* have been roughly categorized into three subfamilies (Figure 4A). Group I and group II (yellow) belong to the *COL* subfamily and feature the CCT domain alongside one or two B-BOX domains. Group III and group IV (red) are classified under the *CMF* subfamily and are characterized by possessing solely a single CCT domain. Lastly, group V (blue) belongs to the *PRR* subfamily, which not only includes the common CCT domain but is also complemented by an additional REC domain. It is important to note that a few genes in groups I, II, and V have partial domain loss (Table S4). From Figure 4B, it can be observed that all *BnaCCTs* contain motif 3 and motif 1. Most *BnaCCTs* in group I contain one motif 2 and one motif 6, while most *BnaCCTs* in group II contain only one motif 2. In group V, most *BnaCCTs* contain one motif 7 and one motif 5. Detailed sequences of these 10 motifs are displayed in Figure S1. Figure 4D shows that most *BnaCCTs* contain one to four introns (eight *BnaCCTs* with a single intron, twenty-two *BnaCCTs* with two introns, twenty-three *BnaCCTs* with three introns, and twenty-one *BnaCCTs* with four introns). The proportion of Bna*CCT*s containing 5–9 introns is relatively low (three *BnaCCTs* with five introns, two *BnaCCTs* with six introns, and only one gene contains seven, eight, or nine introns). Five genes lack introns (*BnaCCT24*, *BnaCCT73*, *BnaCCT77*, *BnaCCT86*, *BnaCCT59*), while *BnaCCT10* has an unusually long intron. In summary, these protein domains are relatively conserved, and the gene structures and conservative motif distributions within the same subfamily are similar.

### 2.4. Cis-Acting Element Analysis of the CCT Gene Family in B. napus

**Figure 5:**
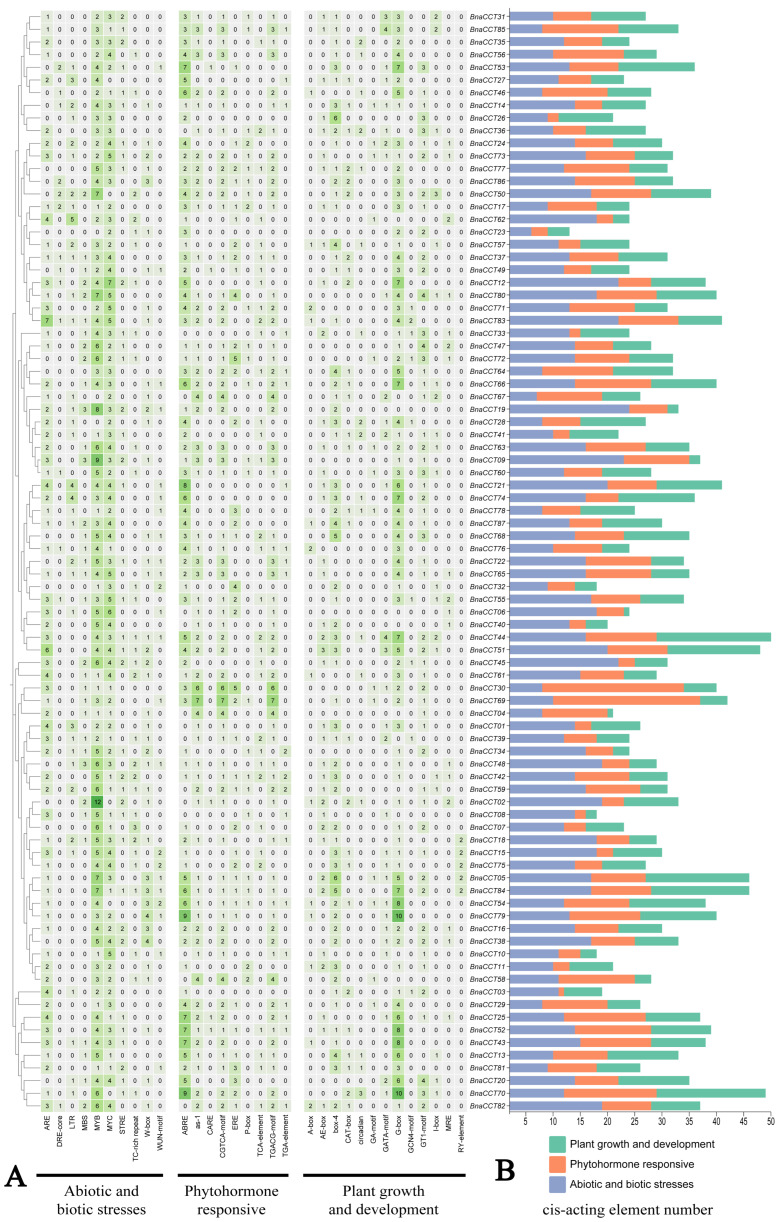
Analysis of cis-regulatory elements in the *CCT* gene family in *B. napus*. (**A**) An evaluation of the distribution of promoter elements across the *BnaCCTs*, where each type is visually differentiated by unique colors and numerical identifiers. This visualization helps to depict the variety and frequency of these elements within the gene family. (**B**) The assessment of the quantity of cis-acting elements, systematically categorized into three distinct functional groups. Each group represents a specific set of roles in the regulatory processes that influence gene expression.

In order to elucidate the regulatory mechanism and stress response of the *BnaCCTs*, an extensive analysis of cis-acting elements within the 2000bp promoter region of the *BnaCCT* family was conducted. Following the exclusion of common cis-acting elements through PlantCARE analysis, a total of 32 cis-acting elements were selected for further examination and visualization using TBtools (Table S5). These cis-acting elements, integral to gene expression modulation, are systematically classified into three distinct categories based on the specific biological functions they influence. The first category encompasses elements that are crucial for mediating responses to both abiotic and biotic stresses, thereby enabling plants to adapt to environmental challenges and pathogenic attacks. The second category includes elements that are responsive to plant hormones, playing pivotal roles in the signaling pathways that regulate various physiological processes. The third category consists of elements that are directly involved in the regulation of plant growth and developmental processes. This classification aids in understanding the complex interactions and regulatory mechanisms that underpin plant survival, adaptation, and growth (Figure 5A). Within the cis-acting elements associated with plant growth and development, a plethora of elements involved in light response, as well as circadian and tissue-specific control, were identified. Notable examples include the G-box, Box-4, GATA-motif, I-box, GT1-motif, and RY-element. 

For hormone-responsive elements, several vital regulatory elements were identified. These elements included several specific regulatory motifs associated with different plant hormones: ABRE, which is crucial for abscisic acid signaling; the auxin response elements, specifically the TGA-element, important for auxin-mediated transcriptional regulation; gibberellin response elements, including the GARE-motif and P-box, vital for gibberellin regulatory processes; the TGA-element associated with indoleacetic acid, another key player in auxin response; the salicylic acid response element (TCA-element), essential for mediating salicylic acid’s role in plant defense mechanisms; and the jasmonic acid response elements, CGTCA-motif and TGACG-motif, which are significant in the jasmonic acid signaling pathway. Among these, the ABREs showed the highest representation. Moreover, various stress-response-related elements, encompassing anaerobic induction (ARE and GC-motif), wound (WUN-motif), and elements involved in general defense and stress reactions, were marked by TC-rich repeats. Furthermore, we identified elements specifically responsive to environmental stressors, such as drought (MBS motifs), high temperatures (STRE motifs), and low temperatures (LTR motifs), highlighting the genetic adaptability of *BnaCCTs* to fluctuating environmental conditions. Notably, the MYB element, identified in 84 out of 87 *BnaCCTs*, was the most abundant cis-acting element within this group, followed closely by the MYC element, found in 82 *BnaCCTs*. The involvement of these two elements in stress response has been widely recognized [28].

### 2.5. Protein Interaction Network Analysis and KEGG Enrichment Analysis of BnaCCTs

**Figure 6:**
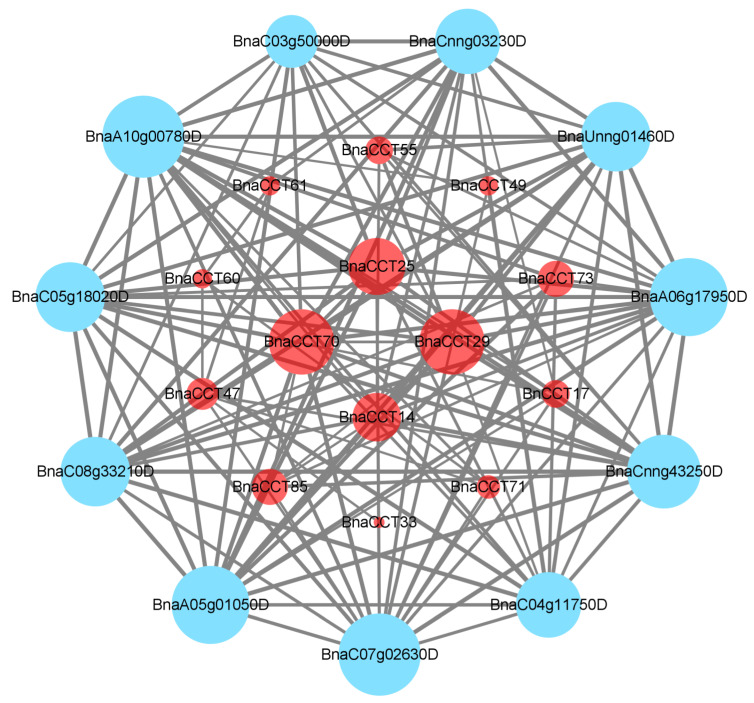
Protein interaction network of *CCT* gene in *B. napus*. The red circle is the *CCT* gene with protein interaction detected by STRING, and the blue circle is the *CCT* gene with protein interaction in *B. napus*.

**Figure 7:**
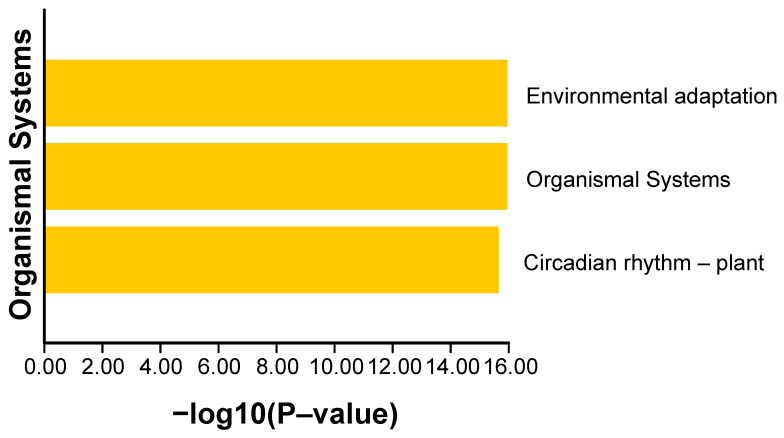
KEGG pathway analysis of *BnaCCTs*.

In order to gain a better understanding of the involvement of the *CCT* gene in various biological processes within *B. napus*, a protein interaction network diagram for the BnCCTs was constructed (Figure 6). The size of each node was determined based on the number of protein–protein interactions (Table S6), thus highlighting that BnaCCT25, BnaCCT70, and BnaCCT29 were annotated as proteins bearing similarity to PRR9, PRR5, and PRR3, respectively. In addition, BnaCCT14, BnaCCT81, BnaCCT55, BnaCCT49, BnaCCT73, BnaCCT17, and BnaCCT71 were annotated as proteins similar to COL9, COL5, COL7, COL3, COL1, COL5, and COL4, while BnaCCT33, BnaCCT85, BnaCCT47, and BnaCCT60 were classified as BBOX17, BBOX13, BBOX14, and BBOX15, respectively. Furthermore, a KEGG pathway map was generated for the set of 87 *BnaCCTs* (Figure 7), which revealed their notable enrichment in pathways associated with environmental adaptation, organismal systems, and circadian rhythm (Table S7). Previous research studies on the *CCT* gene family in *B. napus* have predominantly focused on the Circadian rhythm, particularly on the *CO* gene’s regulation of flowering within the *COL* subfamily [12,22,23,29,30,31]. However, there exists a relatively limited body of research on environmental adaptation. Therefore, the primary objective of this experiment was to center around environmental adaptation in order to explore the response of *BnaCCT*-associated genes to abiotic stress and hormone stimuli.

### 2.6. Analysis of BnaCCT Expression in Various Tissues of B. napus

**Figure 8:**
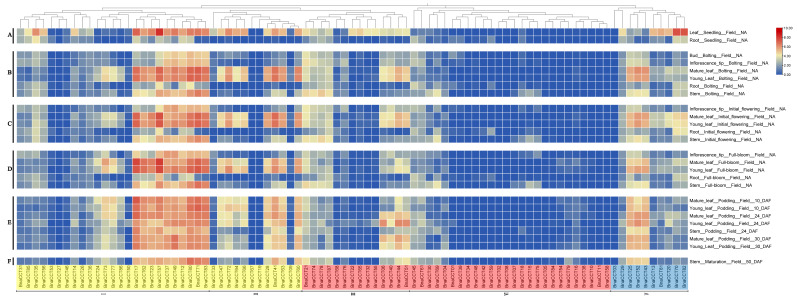
Expression analysis of *BnaCCTs* in *B. napus* cultivar ‘ZS11’. (A–F): The growth stages are denoted as A through F, corresponding to the seedling stage, bolting stage, initial flowering stage, full flowering stage, podding stage, and maturation stage, respectively. (I–V): *BnaCCTs* have been organized into five groups based on a previously established phylogenetic tree.

To analyze the temporal expression profiles of the *BnaCCTs* throughout the developmental phases of *B. napus*, the expression data of 87 *BnaCCT*s (Table S8) were extracted from the BrassicaEDB [32]. Subsequently, a heatmap was generated to display the expression levels of *BnaCCTs* across different tissues and developmental phases, such as roots, stems, young leaves, old leaves, flower buds, and inflorescence tips (Figure 8). The growth stages are denoted as A through F, corresponding to the seedling stage, bolting stage, initial flowering stage, full flowering stage, podding stage, and maturation stage, respectively. Analysis of the heatmap reveals that the expression profiles of the 87 *BnaCCTs* can be systematically classified into three distinct types, each reflecting different regulatory roles these genes may play across the developmental timeline of the plant. The first type comprises genes such as *BnaCCT17*, *BnaCCT62*, *BnaCCT23*, *BnaCCT57*, *BnaCCT37*, *BnaCCT49*, *BnaCCT12*, *BnaCCT80*, *BnaCCT71*, *BnaCCT83*, *BnaCCT29*, *BnaCCT25*, *BnaCCT52*, and *BnaCCT43*, which exhibit prominent expression in multiple tissues. Most of these genes belong to the *COL* subfamily, with a few belonging to the *PRR* subfamily. The second type includes genes belonging to the IV group of the *CMF* subfamily, which demonstrate low expression across most tissues. The third type consists of genes such as *BnaCCT06*, *BnaCCT40*, *BnaCCT44*, and *BnaCCT51*, which exhibit preferential accumulation in leaves as well as genes such as *BnaCCT18*, *BnaCCT15*, and *BnaCCT75*, which were preferentially expressed in stems. These findings indicate distinct expression patterns of *CCTs* across different subfamilies in *B. napus*, with similar expression patterns observed within the same subfamily. Moreover, the significant expression of *BnaCCTs* in leaves compared to other tissues suggests overall higher expression levels in roots as compared to foliage.

### 2.7. Expression Analysis of the PRR Subfamily under Various Abiotic Stress Conditions and ABA Treatment

**Figure 9:**
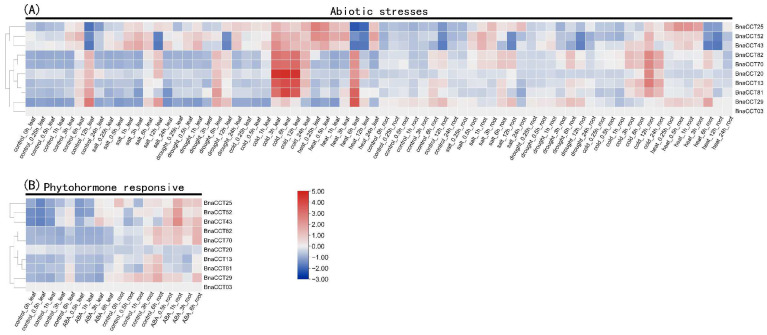
Heat map depicting the response of *PRR* subfamily genes to various abiotic stresses and treatments with ABA hormones. (**A**) The heat map focuses on the expression levels of *PRR* subfamily genes when exposed to various forms of abiotic stress. (**B**) Display of the gene expression levels following treatment with ABA, a key hormone known to mediate plant stress responses.

In our previous investigation of the promoter region of the *BnaCCT*s, we identified specific cis-acting elements and established that the *BnaCCTs* are hypothesized to be critical in mediating a range of physiological and biochemical responses when plants encounter stress. These genes may function as key regulators in various stress-response pathways, facilitating adaptations to environmental challenges. Notably, our protein interaction network analysis revealed that the highly interactive *CCTs* are primarily concentrated within the *PRR* subfamily. Given that previous research has predominantly focused on the *COL* and *CMF* subfamilies in non-biological stress response and hormone-related aspects [19,33,34,35,36,37], and there exists a notable gap in the current scientific literature concerning the role of the *PRR* subfamily in various biological processes within *B. napus*, our study placed particular emphasis on the *PRR* subfamily of the *CCT* gene family in *B. napus*. Consequently, we utilized the BnIR [38] database to examine the expression levels of ten *CCT* genes in the *PRR* subfamily under various stress and hormone conditions (Table S9) and generated a comprehensive heatmap for visualization purposes (Figure 9). Our observations indicated that all *BnaCCTs* within the *PRR* subfamily exhibited distinct expression profiles when subjected to abiotic stress and ABA treatments.

In leaves, the expression levels of all genes were upregulated during the middle to late stages (3–12 h) of cold treatment at 4 °C, with five genes (*BnaCCT81*, *BnaCCT82*, *BnaCCT70*, *BnaCCT13*, *BnaCCT20*) showing significantly increased expression. Notably, *BnaCCT25* exhibited upregulation throughout the entire treatment period. Under salt stress, three genes (*BnaCCT25*, *BnaCCT52*, *BnaCCT43*) showed increased expression in the early stages (0.25–3 h), while *BnaCCT13* and *BnaCCT81* exhibited decreased expression in the middle to late stages. Most genes were insensitive to drought stress except for *BnaCCT52* and *BnaCCT43*, which showed increased expression in most periods. *BnaCCT25* exhibited increased expression at 12–24 h. Under high-temperature treatment at 40 °C, most genes (*BnaCCT29*, *BnaCCT81*, *BnaCCT82*, *BnaCCT70*, *BnaCCT13*, *BnaCCT20*) only showed upregulation during the middle stage (6 h) of treatment, while *BnaCCT25*, *BnaCCT52*, and *BnaCCT43* were downregulated during the same period. Interestingly, these three genes, apart from being downregulated during the middle stage (6 h) of treatment, showed an overall trend of upregulation in response to heat stress. The expression pattern in roots was similar to that in leaves but, in general, qualitatively lower in magnitude.

In leaves subjected to ABA treatment we observed that *BnaCCT25*, *BnaCCT52*, and *BnaCCT43* displayed varying levels of upregulation during the middle stage, whereas the remaining genes showed less pronounced changes. In roots, apart from *BnaCCT13* and *BnaCCT81* which exhibited decreased expression during the middle to late stage (3–6 h), most genes demonstrated a certain degree of upregulation with *BnaCCT25*, *BnaCCT52*, and *BnaCCT43* showing significantly increased expression. It is noteworthy that *BnaCCT03* did not display any detectable expression throughout the entire duration of the abiotic stress and ABA treatments.

### 2.8. PRR Subfamily Amino Acid Sequence Alignment and Prediction of Protein Three-Dimensional Structure

**Figure 10:**
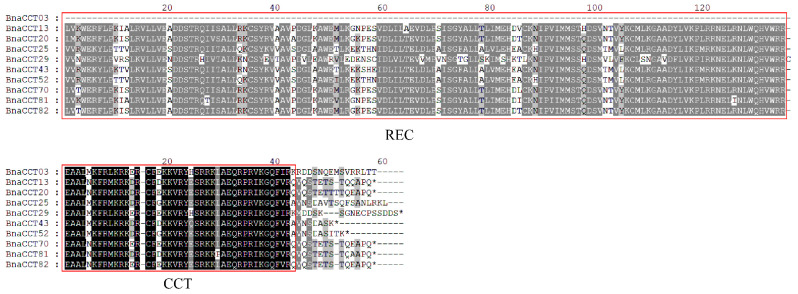
Amino acid sequence alignment of *PRR* subfamily genes. The asterisks denote the end of the entire amino acid sequence.

**Figure 11:**
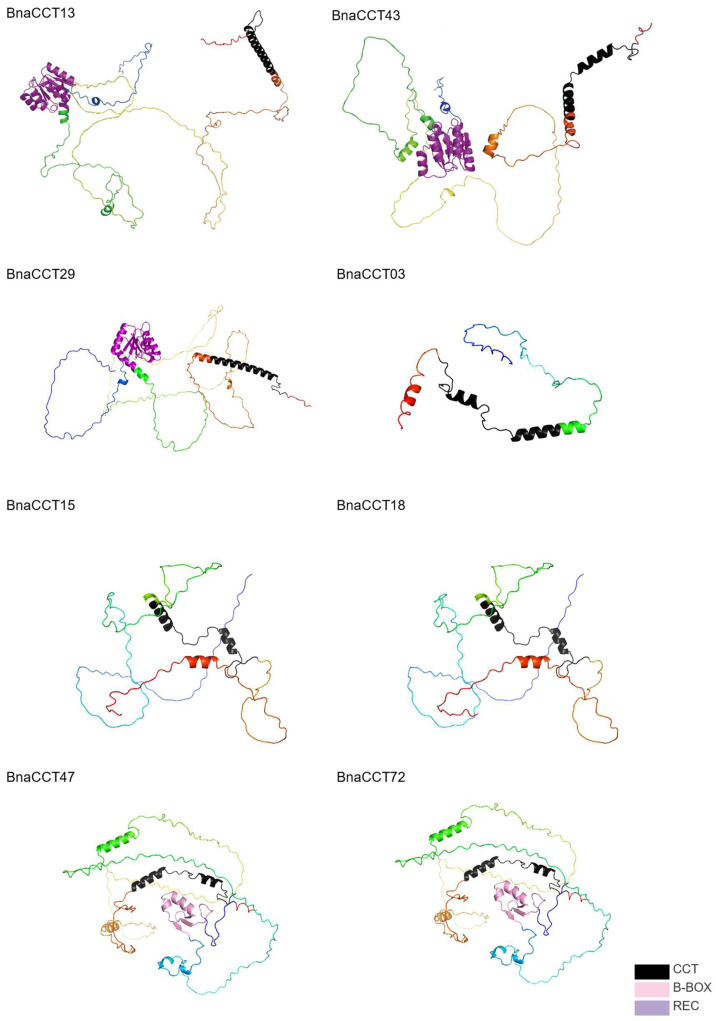
Three-dimensional structures of eight representative BnaCCT proteins.

In the analyses of Section 2.6 and Section 2.7 above, different expression patterns were observed among various subfamilies. The expression patterns within the same subfamily were similar. However, a small number of *BnaCCTs* in the *PRR* subfamily, namely *BnaCCT03*, *BnaCCT25*, *BnaCCT52*, and *BnaCCT43*, exhibited distinct expression patterns compared to other genes under various abiotic stresses and ABA treatments. Amino acid sequence comparisons were conducted for all members of the *PRR* subfamily (Figure 10). The three-dimensional structures of eight representative BnaCCT proteins were predicted, including four members of the *PRR* subfamily (BnaCCT03, BnaCCT13, BnaCCT29, BnaCCT43), two members of the *CMF* subfamily (BnaCCT03, BnaCCT13, BnaCCT15, BnaCCT18), and two members of the *COL* subfamily (BnaCCT47, BnaCCT72) (Figure 11). During the sequence alignments we found that most members of the *PRR* subfamily, except BnaCCT03, possessed two highly conserved structural domains, albeit with some amino acid variations. This may be one of the reasons leading to the differential expression patterns of the *PRR* subfamily genes. Additionally, our protein structure predictions revealed that the three-dimensional structures of proteins from different subfamilies were distinct. Within the *PRR* subfamily, aside from the shared *REC* (purple region) and *CCT* domains (black region), BnaCCT43 contained four α-helices while BnaCCT13 and BnaCCT29 only had two. Furthermore, the lengths and positions of their α-helices differed, which may also contribute to their divergent expression patterns.

### 2.9. qRT-PCR Analysis of the PRR Subfamily under Various Abiotic Stress Conditions and ABA Treatment

In recent years, *B. napus* has been subjected to various abiotic stresses, such as salinity, heat, drought, and cold, resulting in decreased yield and quality. Experimental evidence has shown the significant regulatory role of ABA in plant stress responses [39,40,41,42,43]. Ten genes from the *PRR* subfamily (*BnaCCT03*, *BnaCCT13*, *BnaCCT20*, *BnaCCT25*, *BnaCCT29*, *BnaCCT43*, *BnaCCT52*, *BnaCCT70*, *BnaCCT81*, *BnaCCT82*) and one gene from the *CMF* subfamily (*BnaCCT18*) were selected for qRT-PCR analysis of their expression under different periods (1 h, 1.5 h, 3 h, 6 h, 12 h, 24 h) of various abiotic stresses (salinity, high temperature, drought, low temperature) and ABA treatments in *B. napus* seedling leaves based on previous analyses (Section 2.4, Section 2.5, and Section 2.7). Under 30 μM ABA treatment most *BnaCCTs* showed varying degrees of upregulation, except for *BnaCCT20*, and late stage expression was observed in *BnaCCT25*, *BnaCCT43*, and *BnaCCT52*.

With regard to salt stress responses, the expression patterns of *PRR* subfamily genes under 150 mM NaCl treatment could be broadly categorized into three patterns. The first type comprises five genes (*BnaCCT13*, *BnaCCT20*, *BnaCCT70*, *BnaCCT81*, *BnaCCT82*) with substantial expression during the early and middle stages. The second type consists of three genes (*BnaCCT25*, *BnaCCT43*, *BnaCCT52*) showing significant expression during the later stage. The third type includes *BnaCCT03* and *BnaCCT29*, which exhibited consistently low expression levels throughout the duration. The results suggest that all *BnaCCTs* are involved in the *B. napus* response to salt stress. *BnaCCT25*, *BnaCCT43*, and *BnaCCT52* were primarily upregulated during the late stage of salt stress, while the other genes were expressed during the early phase. Additionally, the *BnaCCT18* gene of the *CMF* subfamily showed minimal to no expression during most periods of salt stress.

Under high-temperature stress of less than 40 °C, all ten *BnaCCTs* in the *PRR* subfamily exhibited varying degrees of expression, falling into three distinct types. The genes *BnaCCT25*, *BnaCCT43*, and *BnaCCT52*, showed high upregulation in both early and late stages. Five genes (*BnaCCT13*, *BnaCCT20*, *BnaCCT70*, *BnaCCT81*, *BnaCCT82*) with moderate expression were mainly expressed in the early to middle stages, peaking at 6 h but, overall, showing less expression than the first type. The third type consisted of *BnaCCT03* and *BnaCCT29*, which exhibited very low expression levels. Under high-temperature stress, the expression of the *CMF* subfamily gene *BnaCCT18* was extremely low. 

To simulate drought stress, we used 20% PEG6000 and observed that all *PRR* subfamily genes, except *BnaCCT03*, were expressed. The expression levels generally followed an early > middle > late pattern. It is worth nothing that *BnaCCT29* showed a certain level of upregulation only at 1.5 h after treatment, with little to no significant change in other phases and may even have been downregulated. The gene *BnaCCT18* of the *CMF* subfamily showed almost no expression during drought stress.

**Figure 12:**
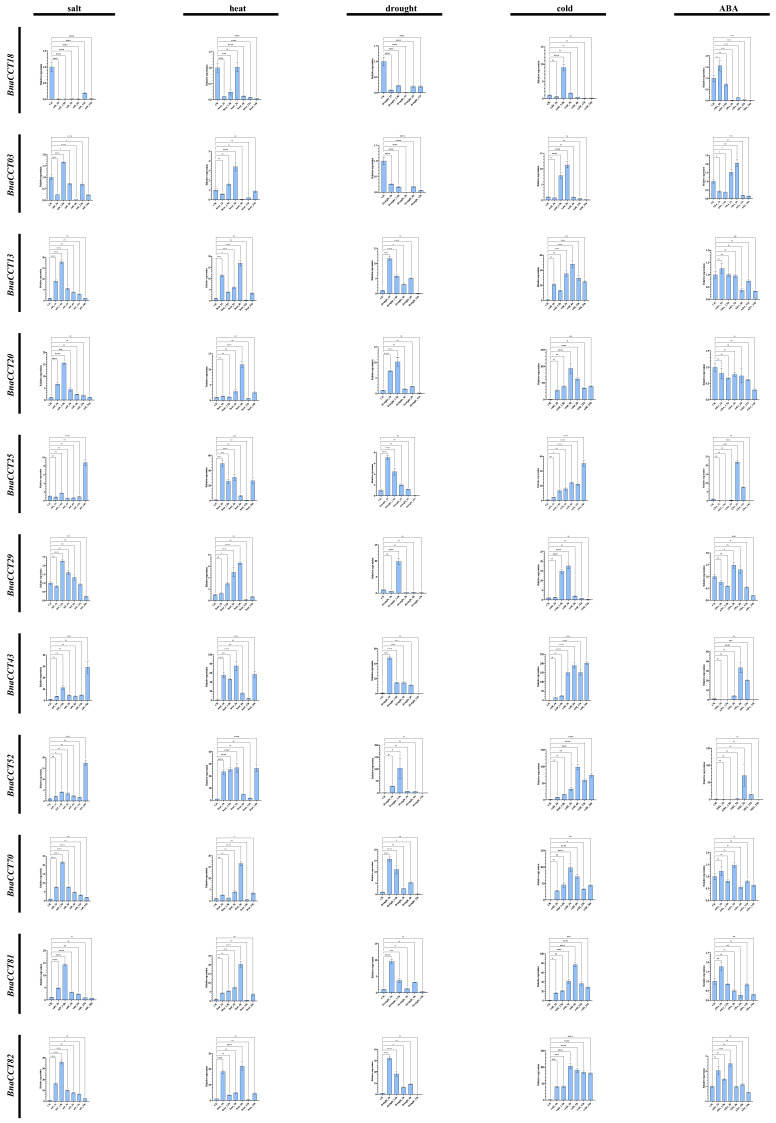
Relative expression of 11 *BnaCCTs* from *B. napus* seedling leaves detected by qRT-PCR. The graphical representation of statistical significance in the figure is denoted using lines and asterisks. The notation “ns” indicates a *p*-value greater than 0.05, while one to four asterisks sequentially signify *p*-values less than 0.05, 0.01, 0.001, and 0.0001, respectively.

During low-temperature stress at 4 °C, all *PRR* subfamily genes were upregulated. Eight genes (*BnaCCT13*, *BnaCCT20*, *BnaCCT25*, *BnaCCT43*, *BnaCCT52*, *BnaCCT70*, *BnaCCT81*, *BnaCCT82*) displayed highly significant upregulation throughout the treatment period while two genes (*BnaCCT03*, *BnaCCT29*) showed significant upregulation primarily in the early stages and a less notable increase in the later stages. The *CMF* subfamily gene *BnaCCT18* also showed a certain degree of upregulation in the early period of low-temperature stress. All qRT-PCR results are already shown in Figure 12.

## 3. Discussion

*B. napus* is widely distributed and extensively cultivated due to its superior yield, oil content, disease resistance, and plant morphology compared to other *Brassica* species, such as *B. rapa* and *B. juncea* [44]. *B. napus* is also one of the main oilseed crops in China. The acquisition of the *B. napus* genome [45] has enabled systematic analysis of its gene family, with many gene families being studied [46,47,48,49]. The *CCT* gene family is a plant-specific gene family that is widely present in many plants. The *CCT* gene family was first described in the *CONSTANS* gene of *A. thaliana* by Putterill et al. in 1995 [8]. Since then, numerous studies have reported its involvement in many important biological processes in various plants [6,7,9,34,35,36,50,51,52,53]. However, research on the *CCT* gene family in *B. napus* has mainly focused on the *COL* subfamily and its regulation of flowering. Currently, there is a gap in the literature concerning how the *CCT* gene family in *B. napus* responds to abiotic stress and ABA hormones. The lack of relevant research somewhat hinders our understanding of the role that the *CCT* gene family plays in the physiological and developmental processes of *B. napus*.

In this study, we identified 87 *BnaCCTs* (Table S1). In the chromosomal localization of *BnaCCTs*, we found that although the distribution of *BnaCCTs* on the chromosomes is not uniform, a total of 35 *BnaCCTs* are present on A2 to A10 and 33 on C1 to C9. The similar distribution across nine chromosomes and the comparable quantities of *BnaCCTs* suggest a strong conservation and crucial functional role in the developmental and evolutionary processes of Brassica napus. This pattern aligns with the behavior of *CCTs* observed in other species, emphasizing consistent evolutionary characteristics [4,19,52]. A phylogenetic analysis of the *BnaCCTs* and *AtCCTs* was conducted, which facilitated the categorization of these genes into five primary groups. Further analysis based on the presence of specific conserved domains allowed us to delineate these groups into three distinct subfamilies (Figure 4A). In the evolutionary tree, multiple *BnaCCTs* were found to be homologous to the reported *AtCCT* genes, such as *BnaCCT29* and *AT5G60100*, and *BnaCCT25* and *AT2G46790*. The high sequence similarity between these genes suggests that the proteins may have similar functions. 

Whole-genome duplication (WGD) is a pivotal factor in the evolutionary advancement of flowering plants. To investigate the evolution of *BnaCCTs*, a collinearity map was constructed between *B. napus*, *B. rapa*, *B. oleracea*, and the model plant *Arabidopsis*. The map identified 56 genes with collinearity among these species (Table S2). The observed collinearity among these genes highlights their stability and conservation throughout evolutionary history, indicating their crucial contributions to the structural and functional development of the *CCT* gene family. Collinearity maps were constructed within the *B. napus* genome (Figure 4), and the Ka and Ks values for 72 gene pairs involved in large segmental duplications were calculated (Supplementary Materials Table S3). We also estimated the timeframe of the occurrence of large segmental duplication events, which led to the diversification of gene function [54].

Research has shown that the emergence of the *CMF* subfamily is attributed to the absence of the B-BOX domain in members of the *COL* subfamily [20]. Therefore, we hypothesize that the emergence of certain *BnaCCTs* may be caused by changes in the domain structure. To investigate this, we conducted an analysis of gene structure, conserved motifs, and structural domains. Our analysis revealed that motifs 1 and 3 jointly constitute the *CCT* domain, as can be seen in Figure 4B,C. All *BnaCCTs* contain the complete domain, which is located closer to the 3′ end. Motif 2 represents the B-BOX domain, while motif 6 predominantly represents the B-BOX domain, except for *BnaCCT26* and *BnaCCT14*, where the B-BOX domain is not evident. The B-BOX domain is located closer to the 5′ end compared to the CCT domain. The REC domain is composed of motif 7 and motif 5. 

Genes grouped within the same subfamily typically share analogous structures and patterns of conserved domains. It is important to note that in groups I and II, *BnaCCT57*, *BnaCCT53*, *BnaCCT50*, *BnaCCT64*, *BnaCCT66*, and *BnaCCT67* lack the B-BOX domain compared to other genes in this subfamily. This absence may be a result of the continuous evolution of *COL* genes leading to the loss of the B-BOX domain. Additionally, in group V, *BnaCCT03* likely lost the REC domain during the prolonged evolution of the *PRR* subfamily, resulting in it containing only the CCT domain. The loss of structural domains may also lead to functional alterations.

Research indicates that the *CCT* gene family responds to photoperiod signals, participates in circadian rhythm regulation, and thus affects plant flowering time [4,8,18,51]. The promoter region of the *BnaCCTs* includes various elements that contribute to light response and circadian rhythm control, such as the G-BOX, I-box, and Sp1. Research on the *CCT* gene family in *B. napus* has concentrated on its role in controlling the timing of plant flowering. Beyond photoperiod regulation, some *CCTs* also have functions related to abiotic stress. A protein interaction network analysis revealed that the BnaCCT proteins with the most interactions are BnaCCT25, BnaCCT70, BnaCCT29, and BnaCCT14. These proteins are, respectively, annotated as similar to PRR9, PRR5, PRR3, and COL9. Previous research has shown that, in *Arabidopsis*, PRR5-VP improves tolerance to cold, drought, and high salt stress by activating DREB1 expression. The strong drought and salt tolerance of PRR5-VP is not related to regulation of flowering time [55]. COL9 has been found to interact with proteins in the nucleus that are linked to stress responses in Arabidopsis. This interaction occurs particularly under conditions of salt or drought stress. The binding of COL9 to these stress-related proteins triggers a cascade of biochemical events leading to the activation of specific genes. These genes are directly involved in mitigating the effects of salt and drought, thereby enabling the plant to adapt to these harsh conditions [56]. Subsequently, it increases the activity of superoxide dismutase (SOD), peroxidase (POD), and proline content to eliminate peroxides and protect cells from damage. Additionally, a study in sand pear found that PpCOL8 and PpCOL9 respond to salicylic acid during pear fruit senescence. Further characterization of PpCOL8 and PpCOL9 during fruit senescence revealed that COL proteins, especially PpCOL8, may interact with PpMADS via the salicylic acid signaling pathway, playing a crucial role in fruit senescence [53]. 

In *apple*, MdBBX37 interacts with JA signaling repressor factors (MdJAZ1 and MdJAZ2), inhibiting the activation transcription of MdCBF1 and MdCBF4, thereby promoting cold tolerance [57]. Moreover, most proteins interacting with *BnaCCTs* are associated with responses to abiotic stress. For instance, BnaA10g00780D is an LHY-like protein, which negatively regulates *soybean* drought tolerance. The expression levels of all four GmLHYs is significantly enhanced under drought conditions, indicating a direct response to environmental stress. In plants where all four GmLHYs have been mutated, there is a substantial increase in drought tolerance. Transcriptomic analysis indicates that GmLHYs are crucial regulators of the abscisic acid (ABA) signaling pathway during drought stress. This regulation suggests that GmLHYs are integral in orchestrating the plant’s adaptive responses to mitigate the effects of drought [58]. 

BnaUnng01460D, annotated as an ELF4-like protein, has been reported to significantly affect the height and stress resistance of *cotton* seedlings when silenced [33]. The *GhELF4* gene may be involved in ABA or light signaling pathways. Additionally, BnaC04g11750D, annotated as a COP1-like protein, has been shown to play a role in salt stress tolerance in *Arabidopsis*. Compared to their wild-type counterparts, COP1 mutants demonstrate enhanced tolerance to salt, which is associated with their increased sucrose content [59]. Furthermore, BnaC07g02630D, annotated as an LKP2-like protein, has been observed to exhibit higher expression levels of the *DREB1A* gene in GFP-LKP2-1 and GFP-LKP2-2 plants compared to two control groups. This indicates that BnaC07g02630D functions not only in response to low-temperature stress but also plays a significant role in drought tolerance. The overexpression of *LKP2* has been shown to enhance the drought tolerance of *A. thaliana* [60]. 

The majority of the annotated proteins play crucial roles in abiotic stress responses, with most interacting *CCT* genes concentrated in the *PRR* subfamily. While research on abiotic stress and hormone responses has predominantly focused on the *COL* and *CMF* subfamilies, there are limited reports on the *PRR* subfamily in *B. napus* regarding non-biological stress and hormone responses [19,33,34,35,36,37]. Therefore, our study primarily focused on the *PRR* subfamily of the *CCT* genes in *B. napus*. Initially, we analyzed the expression profiles of *BnaCCTs* under non-biological stress and hormone treatments using transcriptomic data from BrassicaEDB [32]. Subsequently, validation was performed using qRT-PCR, which indicated that the *BnaCCTs* in the *PRR* subfamily could be broadly classified into three types. This might imply that genes of the *PRR* subfamily exert varying degrees of influence on the abiotic stress tolerance of *B. napus* at different temporal stages.

Based on these results, it is implied that the *BnaCCTs* likely engage in responses to abiotic stresses via mechanisms that resemble those identified in *A. thaliana*. Furthermore, there are distinct expression patterns among different subfamilies, and the functions of these genes have undergone certain differentiation during evolution. It is plausible that the genes in the *PRR* subfamily may have multiple functions.

## 4. Materials and Methods

### 4.1. Identification and Prediction of Physicochemical Properties of BnaCCTs

The whole-genome data and complete protein sequences of *B. napus*, *B. rapa*, and *B. oleracea* were acquired from the BRAD [45] database (http://brassicadb.cn, accessed on 18 March 2023). Additionally, the whole-genome data and complete protein sequences of *A. thaliana* were sourced from the TAIR10 [61] database (ftp.Arabidopsis.org, accessed on 18 March 2023). The hidden Markov model (HMM) of the *CCT* domain (PF06203) was retrieved from the Pfam [24] database (http://pfam.xfam.org/, accessed on 18 March 2023). The first round of domain searches was conducted for *B. napus*, *B. rapa*, and *B. oleracea* using the HMMER program [62] (version 3.1b2) with the same criteria. The candidate *CCT* gene sequences identified in the first round, aligned using Clustal W. Hidden Markov models, were then reconstructed for each species based on the alignment outcomes, followed by a second round of domain searches. Putative members were selected if their E-value was less than 0.001. The prospective member genes’ protein sequences were subsequently subjected to an evaluation using both SMART (http://smart.embl-heidelberg.de/, accessed on 29 March 2023) and NCBI’s Conserved Domain Database (https://www.ncbi.nlm.nih.gov/Structure/bwrpsb/bwrpsb.cgi, accessed on 29 March 2023) to identify their domains. Based on the comprehensive domain and sequence analysis, selections for the *CCTs* in each studied species were finalized. The physicochemical attributes of proteins encoded by the *B. napus CCT* gene were analyzed using TBtools (v2.012) [27]. This included the count of amino acid residues, relative molecular weight, isoelectric point, and hydrophilicity.

### 4.2. Phylogenetic Tree Analysis

Considering the excessive number of *CCTs* among *B. napus*, *A. thaliana*, *B. rapa*, and *B. oleracea*, this study primarily focused on conducting multiple sequence alignment and phylogenetic tree analysis of the full-length protein sequences of the *CCTs* in *B. napus* and *A. thaliana*. The MEGA7.0 (https://mega.software.informer.com/7.0/, accessed on 29 March 2023) was used with the NJ method to construct unrooted phylogenetic trees for the two species, employing a Bootstrap value of 1000 iterations [63]. The unrooted trees were generated based on multiple sequence alignment executed via the Clustal W method in MegAlign (https://www.dnastar.com/software/lasergene/, accessed on 29 March 2023), and its aesthetic enhancement was achieved using ChiPlot (https://www.chiplot.online/, accessed on 29 December 2023) [64].

### 4.3. Analysis of the Genetic Structure, Conserved Motifs, and Structural Domains of the BnaCCTs

The GFF annotation file for the whole genome of *B. napus* was downloaded from the BRAD [45] database. To analyze the conserved motifs of the *CCT* protein in *B. napus* we used the MEME program (Motif Education Multiple Expectation Maximization) [65]. We defined the maximum number of motifs as 10, with lengths ranging from 6 to 200 residues. The conserved domains of the *CCTs* were analyzed using the CDD database. The TBtools (v2.012) software was used to construct the phylogenetic tree, with the predicted results from the MEME program in XML format, and the relevant structural domains filtered from CDD.

### 4.4. Chromosome Localization and Collinearity Analysis

Using the information from the annotated genome of *B. napus*, we located all *CCTs* on their respective chromosomes. To identify tandem repeats and large segmental duplications among *BnaCCTs*, we employed the MCScanX algorithm [66] for collinearity analysis. Visualizations and calculations for the rates of nonsynonymous (Ka), synonymous (Ks), and divergence time (t) were performed using TBtools (v2.012) software.

### 4.5. Cis-Acting Element Analysis and the Prediction of Subcellular Localization

Utilizing the genetic positioning details from the annotation file of *B. napus*, the 2000 bp segment of the *BnaCCTs* family’s promoter was isolated and then uploaded to the Plant CARE server [67] for a thorough analysis of the various cis-acting elements present within it. The resulting data were visualized using the TBtools tool. To predict the subcellular localization of the *BnaCCTs*, Wolf (https://www.genscript.com/wolf-psort.html, accessed on 4 November 2023) [25] was employed.

### 4.6. Protein Interaction Network and KEGG Enrichment Analysis

The full-length protein sequences of *BnaCCTs* were extracted and searched in the STRING database (https://string-db.org/, accessed on 21 October 2023) [68] to explore interacting genes related to *BnaCCTs*. An interaction score threshold of 0.400 was established for relevance, and functional annotations of the interacting genes were queried. Subsequently, a protein–protein interaction network was visualized using Cytoscape (v3.8.2.0) [69] based on the search results. For enrichment analysis, gene functional annotations were performed using eggNOG-mapper (http://eggnog-mapper.embl.de/, accessed on 21 October 2023), and KEGG pathway enrichment analysis was conducted based on the annotation results.

### 4.7. Analysis of CCT Expression in B. napus

We searched for expression data of *BnaCCTs* in various tissues at different growth stages of ZS11, as well as under hormonal and abiotic stress, from the gene expression databases of *Brassica* species, BrassicaEDB (https://brassica.biodb.org/, accessed on 4 November 2023) [32] and BnIR [38] (BnIR, *B. napus* multi-omics database (information resource) (hzau.edu.cn). This was visualized the data using TBtools. 

### 4.8. PRR Subfamily Amino Acid Sequence Alignment and Prediction of Protein Three-Dimensional Structure

To further investigate the reasons for the different expression patterns of *BnaCCTs*, we performed a sequence alignment of the amino acid sequences of the *PRR* subfamily using GeneDoc software (v2.7.000) (https://nrbsc.org/gfx/genedoc/, accessed on 2 January 2024). Additionally, we predicted the three-dimensional protein structures of select representative *BnaCCTs* through SWISS-MODEL (https://swissmodel.expasy.org/, accessed on 18 December 2023), with Pymol (https://pymol.org/, accessed on 18 December 2023) as the visualization tool.

### 4.9. RNA Isolation and Quantitative Real-Time PCR (qRT-PCR)

Ten *PRR* and one *CMF* subfamily members were subjected to various durations (1 h, 1.5 h, 3 h, 6 h, 12 h, 24 h) of abiotic stresses (salt, high temperature, drought, and stress) as well as ABA treatment. RNA was subsequently extracted and reverse transcribed for qRT-PCR analysis, with three biological replicates per treatment. The quantitative polymerase chain reaction (qPCR) was carried out using the ChamQ Universal SYBR qPCR Master Mix, which is a product of Vazyme Biotech, located in Nanjing, China. The qPCR experiments were conducted using the Bio-Rad CFX96, a device manufactured by Bio-Rad Laboratories located in Hercules, California, USA [70]. We adopted the methodologies outlined in the article by Wang to select EV116054 (Table S10) (F-primer: TGGGTTTGCTGGTGACGAT, R-primer: TGCCTAGGACGACCAACAATACT) as our reference gene for the study [71]. The gene expression levels were quantified using the 2^−ΔΔCT^ method, a widely adopted mathematical approach for analyzing the data generated from real-time PCR experiments. The visualization and analysis of the qPCR data were facilitated by GraphPad Prism software (v9.5) [72].

## 5. Conclusions

This study analyzed the *CCT* gene family in *B. napus*. Characteristic analysis was conducted on 87 full-length *BnaCCTs*, which were organized into three subfamilies based on their structural domains. Insights into their collinearity and phylogenetic relationships offer significant information for elucidating the evolutionary dynamics of these genes. In our analyses of cis-acting regulatory elements, protein interaction networks, and KEGG pathway enrichment, we found that the *BnaCCTs*, especially those within the *PRR* subfamily, are significantly involved in enhancing the resistance of *B. napus* to abiotic stresses. Subsequently, we utilized RNA-seq and qRT-PCR analyses to further investigate the specific expression patterns of the PRR subfamily genes, ultimately delineating three distinct expression patterns under abiotic stress and ABA treatment. The first type consists of five genes (*BnaCCT13*, *BnaCCT20*, *BnaCCT70*, *BnaCCT82*) that exhibited upregulated expression in response to most abiotic stresses. The second type comprises three genes (*BnaCCT25*, *BnaCCT43*, *BnaCCT52*) that displayed high expression levels during specific time periods under most abiotic stresses, as well as ABA treatment. Notably, these genes showed significantly greater expression under high temperature, drought, and ABA treatment compared to the other six genes. The third type includes two genes (*BnaCCT03*, *BnaCCT29*) whose expression levels were generally lower than those of the first two types under most abiotic stress conditions. It is noteworthy that *BnaCCT03*, which lacks the REC domain, exhibits a similar expression pattern to certain genes (e.g., *BnaCCT18*) in the *CMF* subfamily. Although the regulatory mechanisms of individual *PRR* subfamily genes remain to be further elucidated, our current study about the expression analysis of *BnaCCTs* in the *PRR* subfamily in response to abiotic stress and ABA treatment offers reference for further research on the expression patterns and functions of *BnaCCTs* related to stress resistance.

## Data Availability

Data used in this study are presented in the Supplementary Materials.

## Supplementary Materials

The following supporting information can be downloaded at: https://www.mdpi.com/article/10.3390/ijms25105301/s1.
