# Supplementary material for: Genome-Wide Identification and Characterization of the CCT Gene Family in Rapeseed (Brassica napus L.)
Source: Int J Mol Sci. 2024 May 13;25(10):5301. doi: 10.3390/ijms25105301 (PMC11121423; doi:10.3390/ijms25105301)
Supplement: Supplementary file 1 [file ijms-25-05301-s001.zip › ijms-2917832-supplementary/ijms-2917832-supplementary/Supplementary Materials/Description of Supplementary Materials.pdf]

Figure 1~12: Figures 1 through 12 are shown in the text; Figure S1: Weblogo plots of the 10 conserved motifs; Table S1: List of *CCT* gens identified from *B. napus*, *A. thaliana*, *B.rapa* and *B. oleracea*; Table S2: Homology of *CCT* genes between *B. napus*, *B.rapa*, *B. oleracea* and *A. thaliana*; Table S3: One-to-one orthologous relationships in *B. napus*; Table S4: The conserved domain of *BnaCCTs* from Batch CD-search tool; Table S5: Major cis-regulatory elements of *BnaCCTs* in *B. napus*; Table S6: Protein interaction network of *BnaCCTs* in *B. napus*; Table S7: KEGG enrichment result; Table S8: The values of *CCT* family genes in *B. napus* by RNA-Seq analysis from BrassicaEDB; Table S9: The values of *CCT* family genes in *B. napus* by RNA-Seq analysis from BnIR; Table S10: Primers used to amplify the *BnaCCTs* and reference genes using qRT-PCR.
