# Supplementary figures and images for: Genome-Wide Identification and Characterization of the CCT Gene Family in Rapeseed (Brassica napus L.)
Source: Int J Mol Sci. 2024 May 13;25(10):5301. doi: 10.3390/ijms25105301 (PMC11121423; doi:10.3390/ijms25105301)

## Motif 1

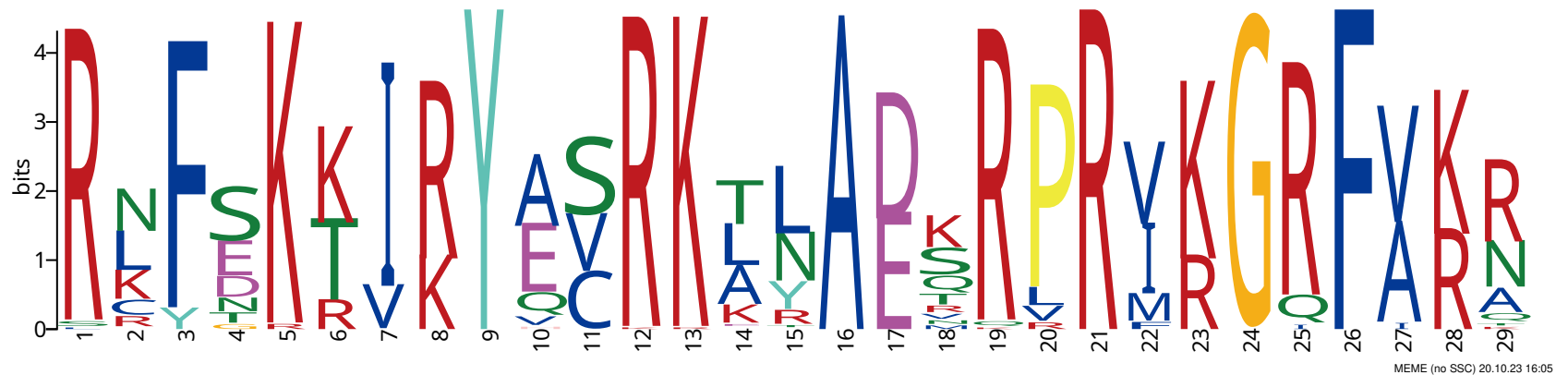

## Motif 2

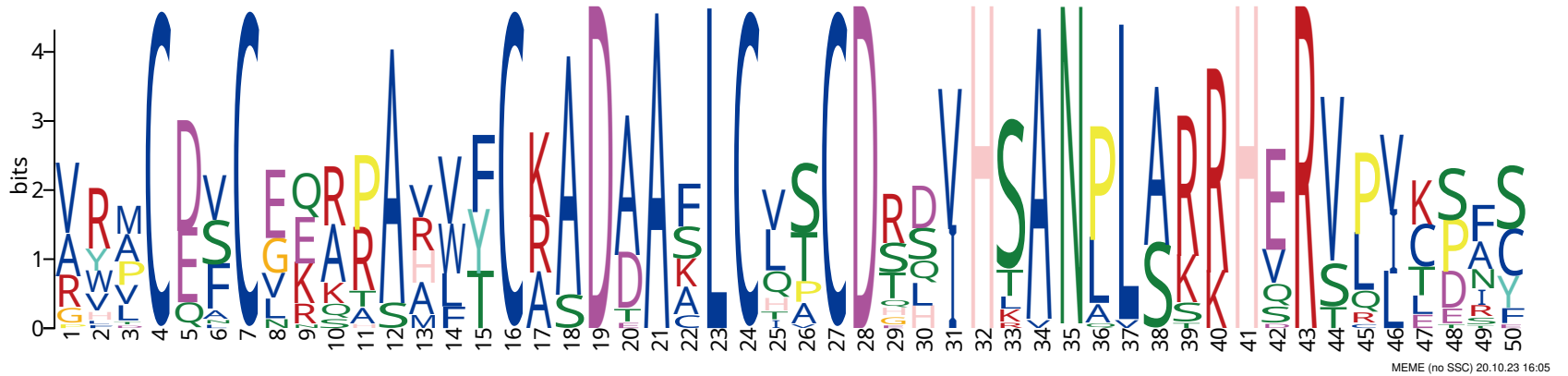

## Motif 3

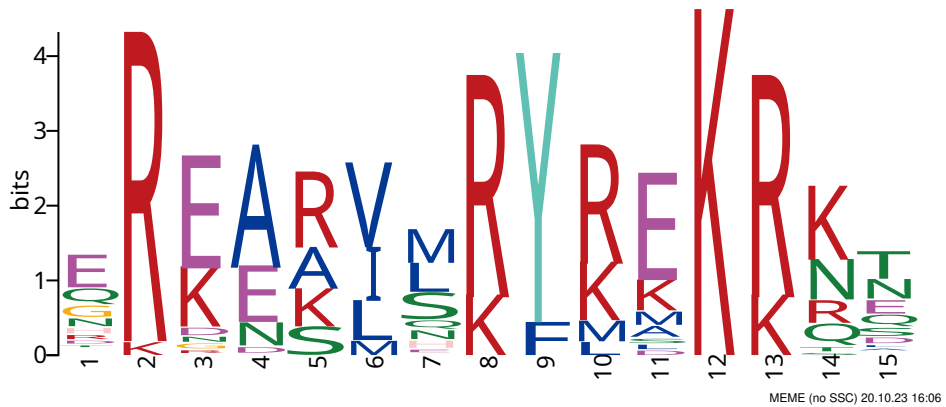

## Motif 4

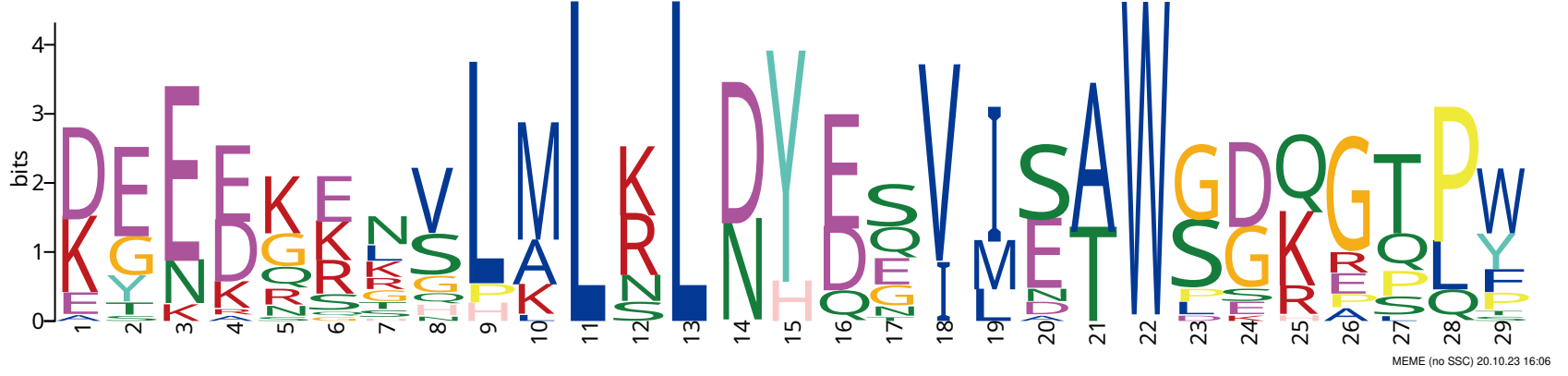

## Motif 5

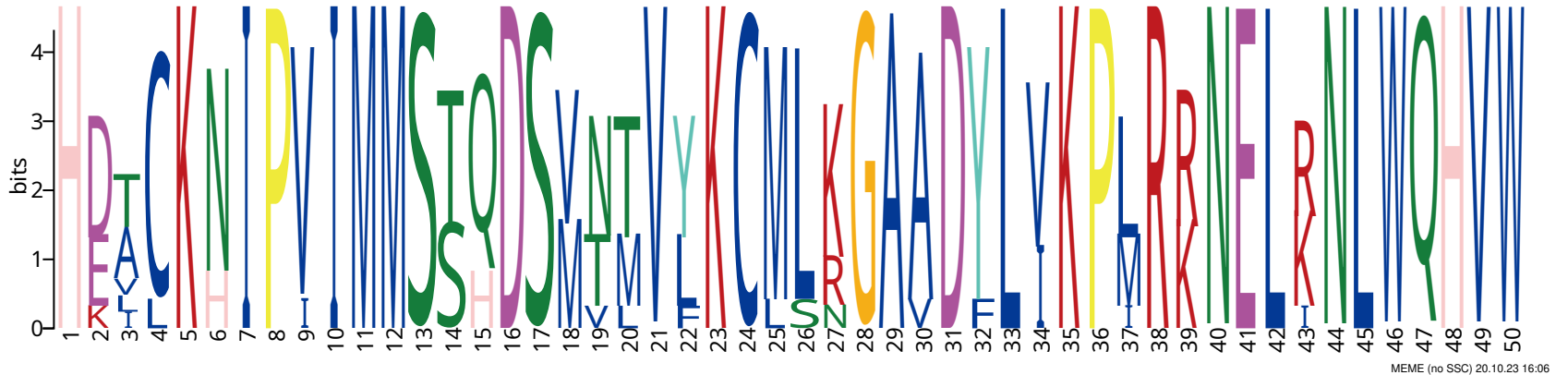

## Motif 6

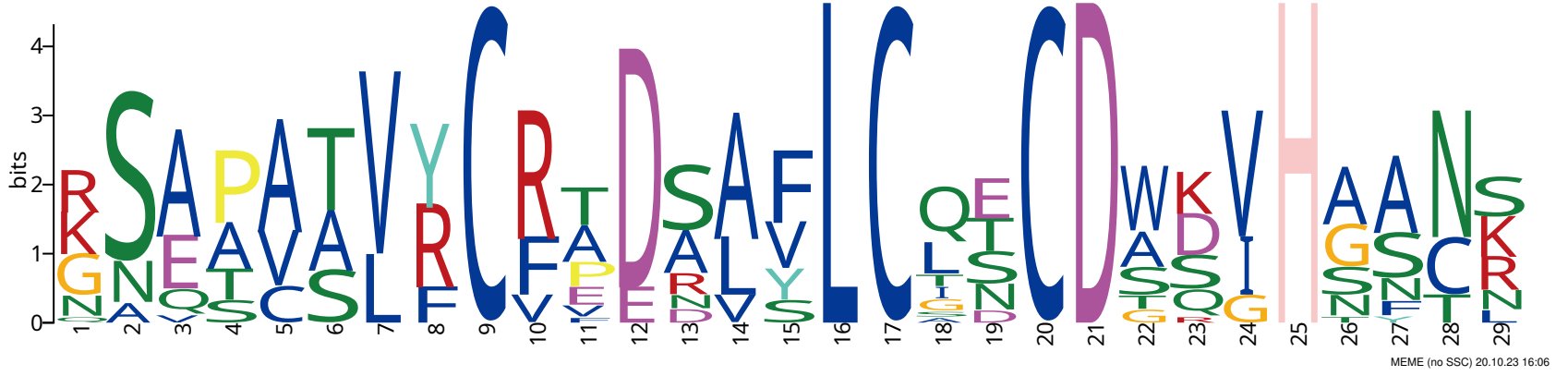

## Motif 7

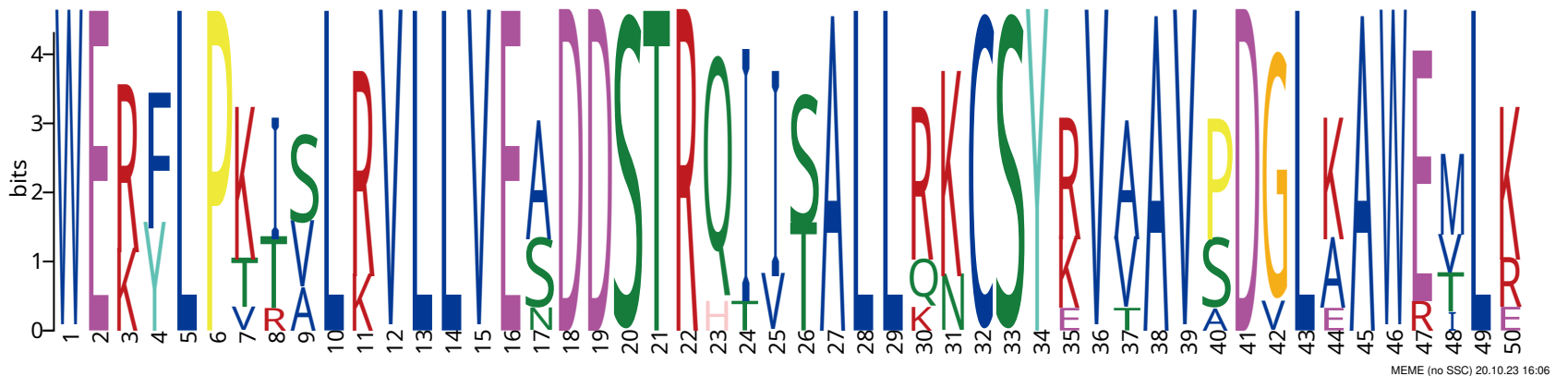

## Motif 8

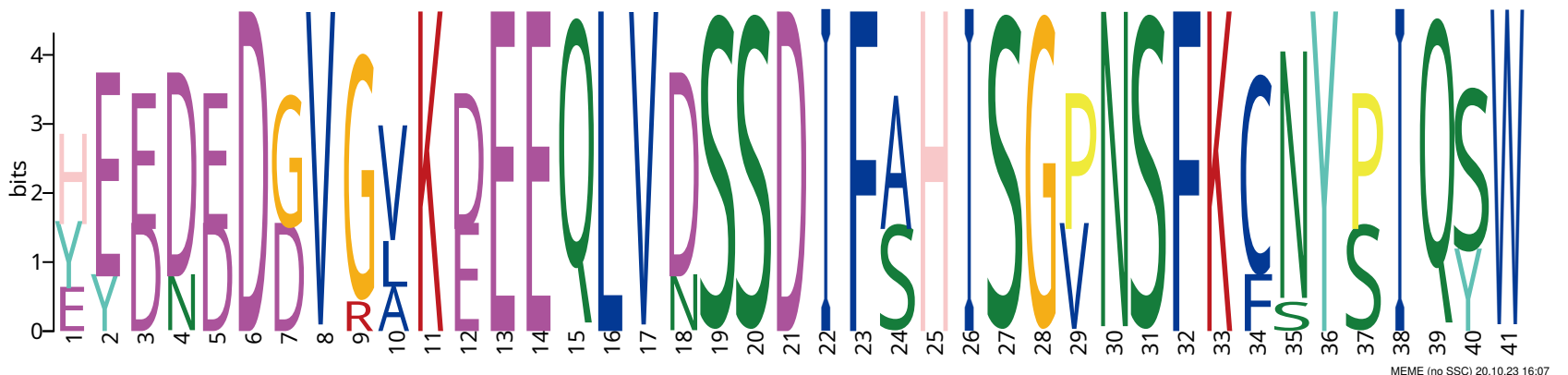

## Motif 9

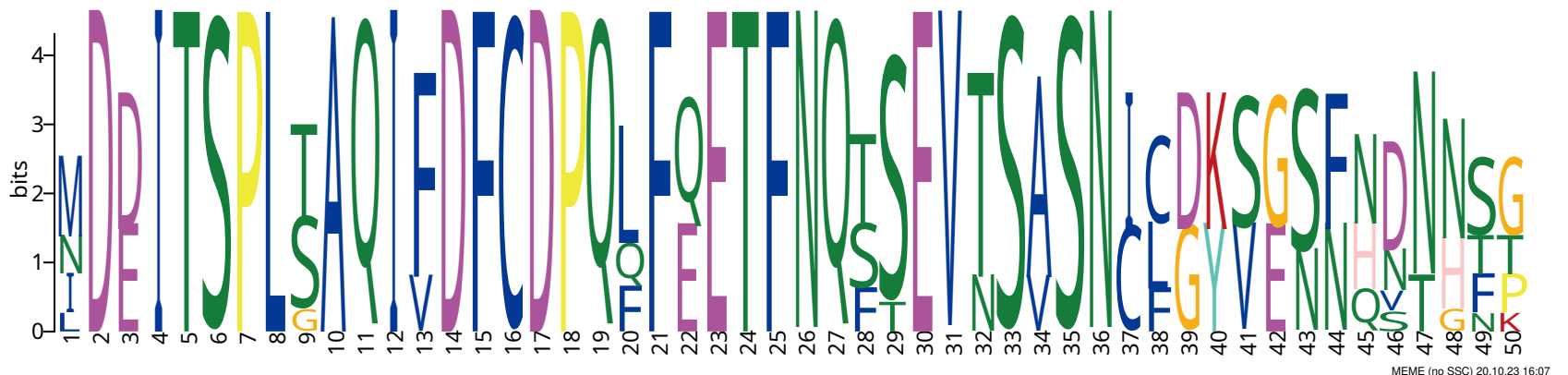

## Motif 1

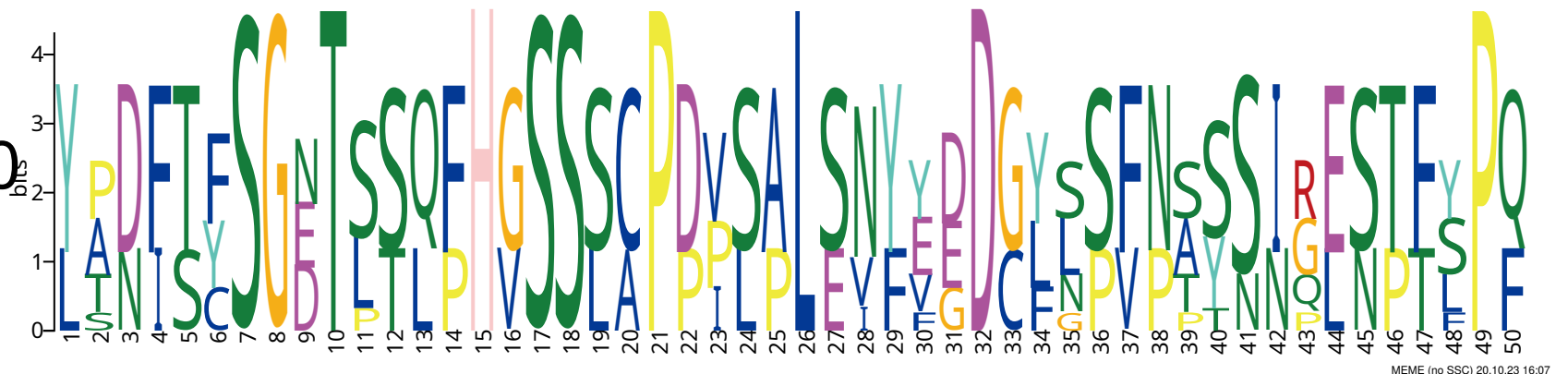

Supplement: Supplementary file 1 [file ijms-25-05301-s001.zip › ijms-2917832-supplementary/ijms-2917832-supplementary/Supplementary Materials/Figure S1.pdf]
